# An ontology-driven tool for structured data acquisition using Web forms

**DOI:** 10.1186/s13326-017-0133-1

**Published:** 2017-08-01

**Authors:** Rafael S. Gonçalves, Samson W. Tu, Csongor I. Nyulas, Michael J. Tierney, Mark A. Musen

**Affiliations:** Stanford Center for Biomedical Informatics Research, Stanford University, Stanford, CA USA

**Keywords:** OWL, Ontology, Structured data, Data acquisition, Form generation

## Abstract

**Background:**

Structured data acquisition is a common task that is widely performed in biomedicine. However, current solutions for this task are far from providing a means to structure data in such a way that it can be automatically employed in decision making (e.g., in our example application domain of clinical functional assessment, for determining eligibility for disability benefits) based on conclusions derived from acquired data (e.g., assessment of impaired motor function). To use data in these settings, we need it structured in a way that can be exploited by automated reasoning systems, for instance, in the Web Ontology Language (OWL); the *de facto* ontology language for the Web.

**Results:**

We tackle the problem of generating Web-based assessment forms from OWL ontologies, and aggregating input gathered through these forms as an ontology of “semantically-enriched” form data that can be queried using an RDF query language, such as SPARQL. We developed an ontology-based structured data acquisition system, which we present through its specific application to the clinical functional assessment domain. We found that data gathered through our system is highly amenable to automatic analysis using queries.

**Conclusions:**

We demonstrated how ontologies can be used to help structuring Web-based forms and to semantically enrich the data elements of the acquired structured data. The ontologies associated with the enriched data elements enable automated inferences and provide a rich vocabulary for performing queries.

## Background

Ontology-based form generation and structured data acquisition was first pioneered almost 30 years ago. In the early 1990s, Protégé-Frames used definitions of classes in an ontology to generate knowledge-acquisition forms, which could be used to acquire instances of ontology classes [1, 2]. The rise of the Web Ontology Language (OWL) [3, 4], standardized by the World Wide Web Consortium (W3C) in 2004, caused a paradigm shift in knowledge representation from frame-based to axiom-based. Because of its axiom-based nature, it is more difficult to acquire instance data based on OWL than it was based on frames. With OWL as the preferred modeling language for ontologies, class definitions are collections of description logic (DL) axioms, and can no longer be seen as templates for forms [5]. Unlike template-based knowledge representations, where what can be said about a class is defined by the slots of the class template, axiom-based representations do not have this kind of locally scoped specification, and allow any axiom describing the same class to be added to the ontology, as long as the axiom does not lead to inconsistencies. Template-based knowledge representation systems use closed-world reasoning and have local constraints (e.g., cardinality of a slot for a particular class) that can be validated easily, while in an axiom-based system with the open-world assumption such local constraint checking is much more problematic. Furthermore, in our chosen application domain, assessment instruments have specific formats that do not lend themselves to be seen as representing instances of domain ontology classes. Items in the instruments have potentially complex descriptions of information to be collected, such as the severity of pain with a particular quality, and at a specific anatomical location. The challenge is to model the assessment instruments and relate the assessed data to a domain ontology with which one can formulate meaningful queries.

In this paper, we describe a system that we developed for representing, acquiring, and querying assessment data that uses: (1) an information model of assessment instruments to drive the generation of data-acquisition Web forms, (2) domain ontologies and standard terminologies to give formal descriptions of entities in our chosen domain, and (3) a data model for the acquired information that links the data to the domain ontologies and standard terminologies. Such linkage makes it possible to query and aggregate the data using the logical representation of the domain concepts in the ontologies. The choice of Web forms as a method for acquiring data is due to their widespread use and simplicity for data acquisition. The form generation software we present here works with forms modeled in OWL so long as these replicate our design pattern for the form specification ontology. The paper describes requirements on the underlying ontologies and information models, and the steps for configuring the software to generate forms and to acquire data using clinical functional assessment as an exemplar.

### Related work

In addition to the comparison with the Protégé-Frames template-based instance acquisition method (in the Background Section), we briefly contrast our work with other systems that use ontologies in the construction of forms for acquiring structured data.

Girardi et al. [6] describe an ontology-based data-acquisition and data-analysis system where the structure of the data depends on the ontology classes, in such a way that the GUI structures (tables, headers, filter dialogs, etc.) can be created at runtime based on the ontology information. This system, like the earlier Protégé-Frames system, assumes that classes in the ontology provide data-acquisition templates that directly define user-interface features. Our system is designed to work with OWL ontologies where ontology axioms do not provide the structural templates required in the system described by Girardi et al. Instead, the structure of the data-acquisition instrument has to be defined separately.

ObTiMA, described by Stenzhorn et al. [7], is another ontology-based data-acquisition system. It is a clinical trial-management application featuring a Trial Builder module that a clinical researcher can use to build case report forms (CRFs). Items in a CRF are constructed by selecting concepts from a master ontology. A Patient Data Management System provides a graphical user interface that allows clinicians to fill in the CRFs relevant to the patient’s current treatment situation. The design of ObTiMA is very similar to the system we are proposing. The main differences, aside from ObTiMA’s specific focus on clinical trial management, include (1) our use of OWL to model not only the domain concepts, but also the structures of forms and data model, and (2) ObTiMA’s use of a tree view to represent concepts that can be selected to define data items. It is understandable that, from the perspective of supporting a clinical researcher’s use of the master ontology to construct CRFs, a tree view provides a necessary simplification of the master ontology, although it nevertheless constrains what can be expressed. It is difficult to see how some of the complex concepts (e.g., ‘constant pain caused by radiculopathy in the lower left extremity’) modeled in our work can be represented as part of a tree structure.

The clinical documentation system developed by Horridge et al. [8] uses a template schema to allow a technology-savvy clinician to create documentation templates that include the local structure of subforms, and potentially complex clinical descriptions consisting of features and their values. The features and values are mapped to a medical ontology, and the system automatically generates ontological descriptions of the data elements based on the mappings. Constrained by our goal to replicate existing forms, we took the opposite approach where we start with ontological descriptions of the data elements, specify how they are used in assessment instruments as part of the description of instruments, and generate forms for the acquisition of data. Having the freedom to design their documentation system, Horridge et al. avoided the laborious work of manually modeling the domain concepts.

Bona et al. developed a work that is similar to ours [9]. They modeled the specifications of forms, question groups, questions, and answers as extensions of the Information Artifact Ontology (IAO),^1^ and the answers as the result of the patient-history taking process. In their work, the questions are just strings that have associated acceptable answers, whereas we attempt to formalize much of the information content in our assessment instruments in terms of a domain ontology. Furthermore, it is not clear that the system automatically generates data-acquisition forms from the ontology-based form specifications.

Outside the domain of biomedicine, semantic wiki is a generic Web-based technology from which one can draw examples on how to arrive at a domain-independent solution. Semantic wikis extend regular wikis with semantic technologies, wherein each wiki article is an RDF resource, and an instance of some resource such as a class defined in the schema,^2^ which can be asserted to have relations with other RDF resources. These relations are defined by the authors of wiki articles, which could be a challenging task to perform without previous knowledge of the domain or the modeling. In a survey of semantic wikis featuring OWL reasoning and SPARQL^3^ querying facilities [10], a user evaluation of a chosen semantic wiki implementation—IkeWiki [11]—concluded that authoring instance data in such a way is cumbersome, even with users that are familiar with ontologies. A good solution to this would be exploiting the relations defined in the schema to provide “wiki article templates” whose form input fields derive from those relations, thus making it easier to create semantic wiki articles – essentially the user would only have to fill in the values of those relations, without having to understand the underlying representation.

Another system that is very close to what we present here is K-Forms [12]. This tool allows users to construct forms using a graphical user interface, and then the resulting form structure is seamlessly encoded as an OWL ontology. However, unlike our work, K-Forms does not have a mechanism to associate form data (whether questions or answers) to user-specified domain ontologies, meaning that the queryability will be constrained to the semantics provided by the system, rather than the more flexible approach that we aim for.

## Implementation

In this section we describe the software, information models, and ontologies that we developed for OWL-based data acquisition.

**Fig. 1:**
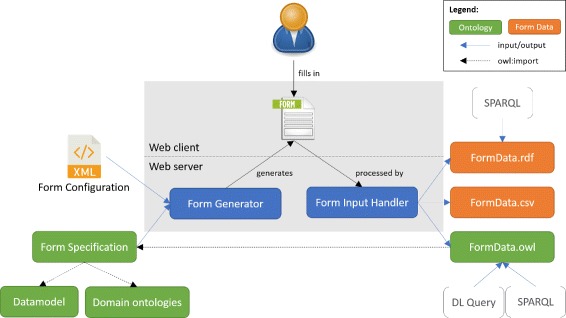
Architecture of the system. The form-generation and data-acquisition software takes an XML configuration file and a form specification as inputs. A form specification uses terms from the *datamodel* ontology to create question instances and to specify possible answers. It annotates questions and answers with concepts from domain ontologies

The architecture of the form generation and data acquisition system we implemented is depicted in Fig. 1. The tool takes as inputs an XML configuration file that specifies the form layout, and a form-specification OWL ontology that defines the content of the form (i.e., the actual questions, answer options, etc.). The tool then generates a form, and outputs answers to form questions in CSV, RDF and OWL formats. We implemented our tool in Java, using the OWL API v4.0.1 [13],^4^ and its source code is publicly available on GitHub.^5^ A Web server is necessary to deploy the application, so the project ships with an embedded instance of Jetty.^6^ The requirements to run the application are Java (v1.7 or above) and Apache Ant.^7^

To try out the software, we supply executable scripts for Windows and UNIX-based operating systems, which build and deploy the tool on the included Jetty Web server. These scripts are hosted in our GitHub repository. First, a user would clone the repository to their computer, and then from a command line execute the appropriate script for the operating system; use ‘run-generator.sh’ on UNIX-based systems and ‘run-generator.bat’ on Windows. Alternatively, one can build the form-generator using Apache Ant, and then deploy it onto the provided instance of Jetty. The application will then be available to browse on the designated localhost port. In addition to the tool itself, we provide in the same GitHub repository 3 example form configurations, the ontologies that we developed, some of the data that we gathered via our tool, example SPARQL queries over that data, and finally the results of executing those queries on our data. Users can open the output data and query it with the example SPARQL queries we supply, using, for example, the Protégé ontology editor [2].

The two major stages in the application workflow are: form generation and form input handling, as described below.

1. Form generation – Steps to produce a form:
  - Process XML configuration file, gathering form layout information, IRIs and bindings to ontology entities
  - Extract from the input form specification ontology all relevant information pertaining to each form element:
    1. Text to be displayed (e.g., section header, question text)
    2. Options and their corresponding text, where applicable
    3. The focus of each question
  - Generate the appropriate HTML and JavaScript code
2. Form input handling – Once the form is filled in and submitted:
  - Process answer data and create appropriate individuals
  - Produce a partonomy of the individuals created in (2.a) that mirrors the layout structure given in the configuration
  - Return the (structured) answers to the user in a chosen format

An application can combine the data with the OWL ontologies to make description logic queries that interpret the data in terms of the semantics defined in the ontologies.

In order to use our tool, a user will have to model questions and their descriptions in OWL, and then specify the layout and content of the resulting form in an XML file. In the following subsections, we will describe the OWL modeling and configuration components in detail.

### Modeling

Our goal was to develop a set of light-weight ontologies and models with minimal ontological commitments, and postponing alignment with possible upper-level ontologies to the future. Existing ontologies, such as the Information Artifact Ontology, do not provide a modeling of forms and questions that we could reuse. Furthermore, what we need is an information model that states, for example, that the structure of a question on a form includes a specific text string, not an ontology that characterizes parts of information artifacts in terms of logical descriptions (e.g., modeling the text of a question as an instance of “textual entity" class).

The modeling component of our software consists of (1) a *datamodel* that specifies the structure of data-acquisition forms and of the resultant data, (2) *form specifications* that define specific data-acquisition forms in terms of the datamodel structures and concepts and relations in the domain ontologies, and (3) one or more *domain ontologies* that define the concepts and relations in an application domain. The domain ontologies that we developed are hosted and maintained in our GitHub repository.^8^

#### Datamodel

**Fig. 2:**
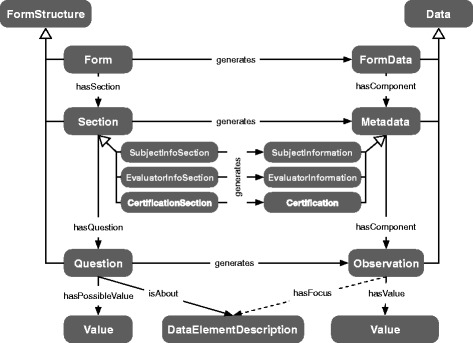
Modeling of the *datamodel* ontology. Excerpt of ontology classes and the relations between these. The classes on the left of the diagram are kinds of *FormStructure*, such as *Question*, which generate instances of the corresponding *Data* classes on the right (e.g., an answer to a question generates an instance of *Observation*)

The *datamodel*, represented as an OWL ontology, is a generic, context-free description of the information structures of a form. It models form elements such as sections and questions, and the data elements generated from a form (e.g., a string value from a text area, or values from an enumerated value set). Figure 2 summarizes key aspects of our modeling: elements of a form are asserted as subclasses of *FormStructure*, such as *Form*, *Section* and *Question*. Each kind of *FormStructure* generates some kind of *Data*; every form submission generates an instance of *FormData*, which references (via the *hasComponent* property) all instances of *Data* generated in the process of parsing form answers. Specific sections such as *SubjectInfoSection* collect information pertaining to a subject, and these details are aggregated in an instance of *SubjectInformation*. An answer to an instance of *Question* gives rise to an instance of *Observation* with a *hasValue* property assertion to the IRI of the selected answer. An instance of *Observation* will be inferred to have an outgoing *hasFocus* property assertion if the *Question* instance it derives from encodes some kind of semantic description of the question’s meaning via the *isAbout* relation. The semantic description is defined as *DataElementDescription* in the domain ontologies. Each instance of *Question* specifies a set of possible (answer) values via a *hasPossibleValue* relation to an instance of *Value*.

#### Form specifications

The *form specifications* contain the sets of OWL individuals that define the content of forms to be generated. Each such form specification contains instances of *Question*, *Section*, *Form* and other elements defined in the *datamodel* ontology (shown in Fig. 2). Not only does the form specification rely on *datamodel* (for form structuring purposes), it also relies on classes and individuals of domain ontologies for semantic descriptions of what the question “is about” (e.g., severity of pain in a body part), and for answer options to the question (e.g., values of a scale of severity of pain).

#### Domain ontologies

These ontologies should provide the classes *DataElementDescription* and *Value*, which are the key links between domain elements and the questions and answers in a form. A developer is free to create subclasses of *DataElementDescription* and *Value*. A question in a form specification may have an *isAbout* property assertion whose filler is an individual of *DataElementDescription*. The semantics of the individual would typically be described by a class expression involving various domain classes. In the domain of functional assessment that motivates our work, a question may be about some attribute of a body function of a person’s body part. If the question is about the severity of the pain sensation in the left lower leg, that question can be annotated with an individual composed as follows (1) assessed function “pain in body part”, (2) anatomical location “lower extremity” with laterality “left”, and (3) assessed attribute “severity”. To formalize the composition, the domain ontology would, at minimum, include classes for body functions, anatomical locations, and assessment attributes. Additional descriptors would require other ontology components.

The granularity of the *isAbout* relation can be as coarse or as fine as necessary. In the aforementioned example, we may decide to leave out the laterality descriptor of a body part. Questions about pain in the right and left lower extremity will have the same *isAbout* relation. In this case, queries that rely on the structures of the domain ontology will not be able to distinguish between answers about right and left extremities. That may be sufficient for the purpose of the user application. As is often the case, we have to balance the precision of allowed queries with the required modeling effort.

### Configuration File for Form Generation and Data Acquisition

The user-defined XML configuration file that drives the form generation process specifies: input and output information of the tool, bindings to ontology entities, and layout of form elements. A Document Type Definition (DTD) schema defines the building blocks of such configuration files, imposing necessary constraints to ensure the configuration file can be suitably interpreted. The key XML elements to be configured by potential users are: *input:* contains an *ontology* child element, and optionally a child element *imports*

- ∘ ***ontology:*** absolute path or IRI to the form specification ontology
- ∘ ***imports:*** contains *ontology* child elements, which have an attribute *iri*, giving the IRI of the imported ontology

*output:* contains the following child elements

- ∘ ***file:*** defines, via a *title* attribute, the title of the form. Optionally, a path can be specified within the *file* element pointing to where the HTML form file should be serialized to
- ∘ ***cssStyle:*** the CSS style class to be used in the output HTML

*bindings:* defines mappings to ontology entities, such as what data property is used to state the text of a question, or section headings *form:* defines the layout and behaviors of the form, as described below

There is a wide range of versatility when configuring forms, such as: multiple levels of sub-questions, form element numbering, question type (e.g., radio, checkbox, dropdown, horizontal checkbox, etc.), question-list layout (vertical or inline) and recurrence; one can specify that a collection of questions should be repeated any given number of times. Some more complex options include overriding the default (alphabetic) order of answer options, and triggering sub-questions when a specific answer is selected. The tool collects the list of answer options for each question from the ontology, and by default this list is ordered alphabetically for presentation. In a situation where there is a need to sort answer options differently, one can do so in the configuration by specifying an attribute *optionOrder* for the question.

Within the *form* configuration element, we start by specifying the IRI of the OWL individual which represents the form, by means of an *iri* element; this is done so that one can, for example, analyze different submissions of the same form. Next we define *section* elements: each *section* element has an *iri* child element with an IRI corresponding to an instance of the *Section* class in the form specification ontology. One can specify the type of section, and whether it is numbered, via *type* and *numbered* attributes, accordingly (see Table 1).

**Table 1:** Acceptable attributes on *section* elements

Name	Accepted values	Description
*type*	question_section	Normal question section or
	subject_section	information aggregation-type sections
	evaluator_section	
*numbered*	true/false	Whether this element should
		be numbered

In a *section* we can have two types of lists: *questionList* and *infoList* (distinguished in the paragraphs below). The way in which either of these lists are presented on the form can be modified via a *type* attribute, which accepts ‘inline’ (horizontally laid out) and ‘normal’ (vertically laid out) types, as shown in Table 2. One can also state in a *repeat* attribute how many times the list should be repeated, which is particularly useful when requesting the same kind of information multiple times, for example, patient symptoms, or previously diagnosed medical conditions.

**Table 2:** Acceptable attributes on *questionList* and *infoList* elements

*Name*	*Accepted values*	*Description*
*type*	inline/normal	Present list elements horizontally (inline) or
		vertically (normal)
*repeat*	positive integer	Repeats the elements within this list the
		specified number of times

The typical output of forms is a list of key-value pairs, i.e., the form output format enforces a one-to-one correspondence between questions and answers. Our tool supports this format with the *questionList* element. However, there are situations where multiple questions in a form represent different aspects of the same entity. For example, personal information fields such as name, phone number, address, etc., are used to represent a single entity: a person. In such cases, we would like to be able to specify that a section of a form is specifically dedicated to gathering multiple details about the same thing. So, in addition to the typical one-to-one format, we support a special kind of list, called *infoList*, that groups multiple answers about the same entity.

Each answer given to a *question* in a *questionList* results in a single instance of *Observation* being created, that is, there is a one-to-one correspondence between input given to a *question* element and an instance of data. Inside *questionList* we can have multiple *question* elements, each with a child *iri* element pointing to the OWL individual representing the question. A question can have sub-questions, which are listed in a *questionList* element within the *question* element. The attributes allowed on *question* elements are specified in Table 3.

**Table 3:** Acceptable attributes on *question* elements

*Name*	*Accepted values*	*Description*
*type*	text	The type of desired HTML form input element, if any; when ‘none’ is selected, no
	textarea	input field is generated. The ‘checkbox-horizontal’ is a variant of the ‘checkbox’
	radio	where options are presented horizontally
	checkbox	
	checkbox-horizontal	
	dropdown	
	none	
*numbered*	true/false	Whether this element should be numbered
*required*	true/false	Whether an answer to this question is required
*optionOrder*	semicolon-separated	Specifies the order in which options are presented. The symbol “*” stands for
	positive integers and “*”	“the remaining, non-listed options”
*showSubquestions For Answer*	Answer IRI	Sub-questions are initially hidden. When the specified answer is selected the
		sub-questions appear
*hideSubquestions For Answer*	Answer IRI	Sub-questions are initially visible. When the specified answer is selected the
		sub-questions disappear

In Table 3 we show the attributes for the key element that users configuring forms would have to define. One of those attributes is to modify the default—alphabetical—order in which answer options are displayed in the resulting form. For instance, we can say that the 4th option should be presented as the last one, like such: optionOrder=“*;4”. To extend on that, we can also have the second element as the first: optionOrder=“2;*;4”.

Answers given to elements inside an *infoList* are aggregated in order to describe a single OWL individual. Within *infoList* elements one can specify multiple *info* elements. The input entered into an *info* element becomes the filler of a data property assertion on the individual specified by the list. The IRI of the data property to be used in each *info* element is given as a *property* attribute on the *info* element (see Table 4).

**Table 4:** Acceptable attributes on *info* elements in addition to the *type*, *required*, and *optionOrder* attributes, defined in Table 3, which can also be used for *info* elements

*Name*	*Accepted values*	*Description*
*property*	Data property IRI	The IRI of the data property which binds
		this element’s input to the individual
		specified in the surrounding infoList

### Application Domain

Clinical functional assessment provides both the motivation for our work, and a use case for our system. Functional assessment is the evaluation of an individual’s ability to perform body functions (e.g., flexing a joint) and defined tasks (e.g., walking a specific distance). It is necessary for evaluating disabilities for rehabilitation, for social security payment, or for decisions to retain or discharge service members who may be injured on duty. Despite its importance, it is not usually supported by electronic health record (EHR) systems [14]. These assessments are often documented using assessment instruments (e.g., check-lists and validated questionnaires) such as Karnofsky Performance Status [15]. Too frequently the data derived from using these instruments are saved as either blobs or non-standard data elements. While a standard such as LOINC®; (Logical Observation Identifiers Names and Codes) defines the syntactic structures of assessment instruments as a hierarchy of panels with questions that have coded answers [16], it does not relate the semantic content of the questions and answers to standard terminologies and data models that allow meaningful querying and aggregation of acquired data.

In order to capture the semantic distinctions that are needed in functional assessment, we developed a Clinical Functional Assessment (CFA) ontology that models the concepts and relationships that occur in functional assessment instruments. Our ontologies reference the International Classification of Functioning, Disability and Health (ICF),^9^ developed by the World Health Organization (WHO), and other reference terminologies such as SNOMED CT.^10^

**CFA** The Clinical Functional Assessment (CFA) ontology models concepts and relationships that allow us to give formal descriptions of the findings, assessments, and measurements embodied in clinical functional assessment instruments. The ontology is divided into three main branches: (1) *Finding*: the entity that is the subject of an observation or judgement, a subclass of the *DataElementDescription* that is required for annotating form questions, (2) *Value* that defines collections of possible qualifiers and values for findings, and (3) *SubjectMatterOntology* that provides internally defined domain concepts that are either not available from standard terminologies, or are references to standard terms that need to be organized into taxonomies. The *Finding* class is further subdivided into *Assessment* (those findings that have non-numeric result) and *Measurement* (those findings that have numeric results). We also define *FunctionalFinding* (a subclass of *Finding*) and *FunctionalAssessment* (a subclass of *Assessment*). In general, a functional assessment will have some assessed function that can be related to an ICF body function or activity (possibly as an exact match, specialization, or generalization), some assessed attribute, such as severity, that specifies the dimension of the function being assessed, and, optionally, some anatomical location of the assessment. Both findings and functions can be modified by qualifiers that further refine these entities. For example, a functional assessment may be made in the context of using assistive devices, and a function being assessed may have some temporal component (e.g., constant or intermittent pain). ICF being an imported ontology for CFA, all ICF categories, such as body structure, body function, activities and participation, and environmental factors are available for formalizing descriptions of functional assessments. For other standard terminologies such as SNOMED CT, ICD, and LOINC, instead of importing them as ontologies, we make references to them through an *ExternallyCodedValue* that specifies the terminology source and code. Queries that reference these codes require the availability of terminology services that relate these codes to other terms in the referenced terminologies.

**Fig. 3:**
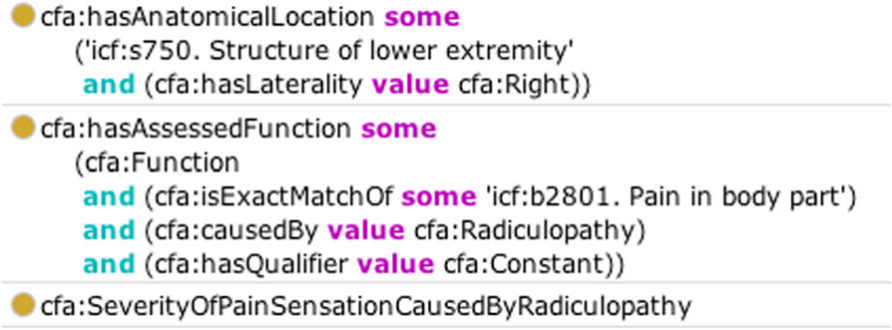
Modeling of the concept of “severity of constant pain caused by radiculopathy in the lower right extremity”. Example modeling from the CFA ontology, which specifies the anatomical location of the symptom (first axiom), and the function being assessed (second axiom)

The modeling of *Finding* is exemplified as follows, based on the “Back (Thoracolumbar Spine) Conditions” DBQ^11^ that we use as one of our exemplar assessment instruments; in the question on the severity of constant pain caused by radiculopathy^12^ on the right lower extremity, we define a subclass of *FunctionalAssessment* that has the assessed attribute ‘severity’, the assessed function ‘icf:b2801 Pain in body part’ that is qualified by a temporal quality ‘Constant’, and has anatomical location ‘icf:s750. structure of lower extremity’ with laterality ‘Right’. Figure 3 illustrates the modeling of this assessment. With the modeling of the dimensions of assessment instrument questions, we can make queries on, and aggregate data collected through the instruments, as will be shown in the Analyzing DBQ Submission Data Section.

#### Form Specifications

**Fig. 4:**
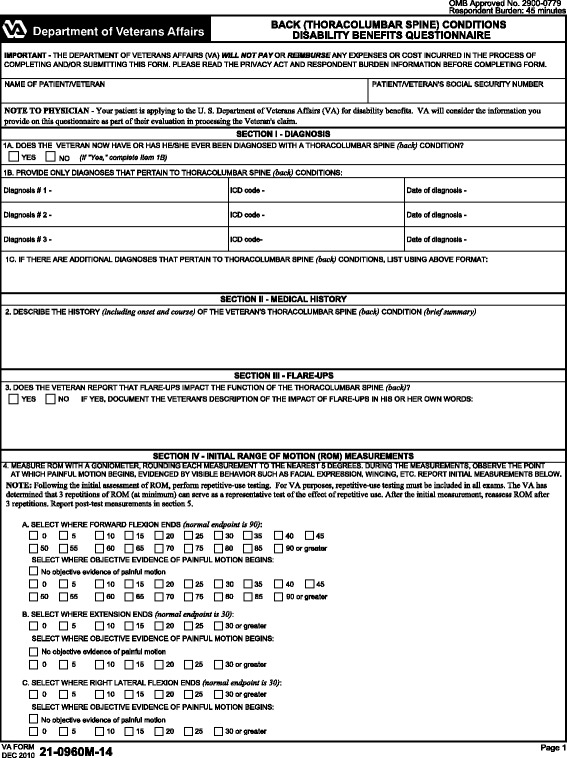
Original VA DBQ. An example DBQ for assessing back (thoracolumbar spine) conditions in PDF format, as distributed by the VA

In our application scenario we use, as exemplars, the U.S. Department of Veterans Affairs (VA) Disability Benefits Questionnaires (DBQs). The top section of one such questionnaire is shown in Fig. 4. DBQs are used to evaluate service members’ disabilities and to determine the benefits for which they are eligible. We start off with these DBQs as our initial form specifications, and design an ontology-based method for Web form generation and structured data acquisition, subsequently exemplifying how one would go about exploiting such data for immediate or *post facto* analyses.

## Results

The key output of the data acquisition tool is the OWL ontology, as it provides us with “semantically enriched” form data that can be used for aggregation and querying. The resulting data individuals are structured in OWL (via *hasComponent* relations) similarly to how the form is structured in the configuration, that is, if question *Q* is configured as having two sub-questions, then the *Observation* individual generated by *Q* will have two outgoing *hasComponent* relations to the instances of *Observation* generated by the two sub-questions of *Q*. The output ontology is modeled according to the *datamodel* ontology presented in the Modeling Section, which is a resource of the overall system distribution.

We configured the complete VA DBQ for ‘Back (Thoracolumbar Spine) Conditions’ using our form generation system, together with the ontologies presented in the Implementation Section—this process is described in the Generating Web-based DBQs Section. The generated Web-based DBQ was validated in an iterative manner by one of the authors (Michael J. Tierney), and subsequently filled in with sample data that we analyze in the Analyzing DBQ Submission Data Section.

**Fig. 5:**
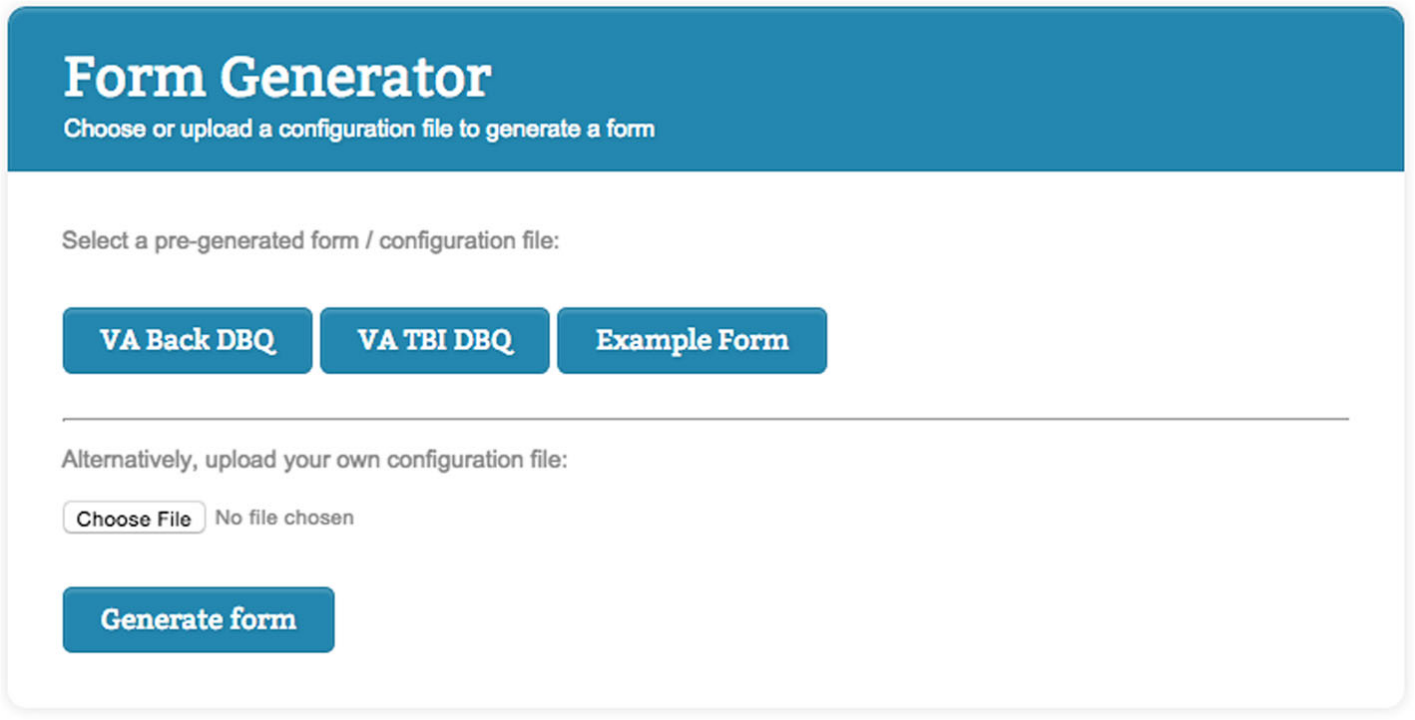
Front page of the form generator. User interface of the front page of the form generator, where users can choose what form to fill in from a list of pre-generated forms, or upload their own configuration file for generating a form

Our pre-configured DBQ forms, as well as an example form which aims at demonstrating all possible options to lay out and configure forms, are shown in the main interface of the form generator, as illustrated in Fig. 5.

### Generating Web-based DBQs

**Fig. 6:**
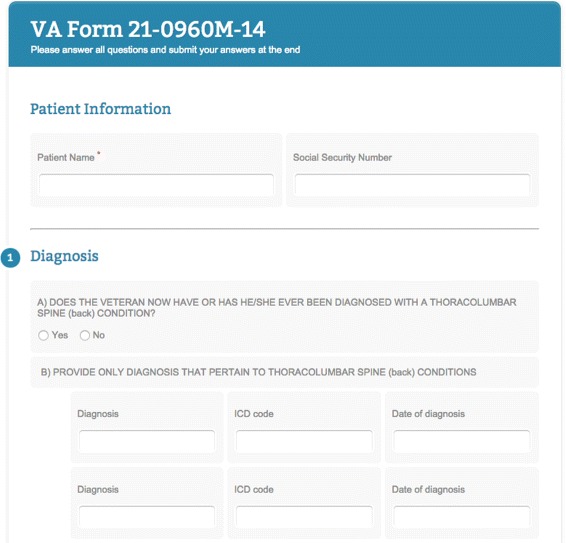
Top portion of the generated Web-based DBQ form for back conditions. The form contains two visible sections: (1) a patient information section, whose answers (name and social security) become data property assertions of the patient individual, and (2) a question section, consisting of a radio-type question and an inline, repeating question list containing 3 text input questions



To generate a “VA Back DBQ”, the corresponding XML configuration file is passed on to the service, which returns the HTML form—the top portion of the resulting form is presented in Fig. 6. Taking into account the elements in Fig. 6, we now inspect how they were specified in the XML configuration (fragments of which are in Code Snippet 1). From top to bottom of Fig. 6 we have: a non-numbered patient-information section (see Line 1 of Code Snippet 1) and a numbered question-section of the DBQ (*section* element without attributes). In the patient information section we have an inline information list (Line 2) with two information inputs: a required “Patient Name” input (Lines 3-4), and an optional text input (no *required* attribute) which gives us fillers of the *cfa:hasID* data property. In the question section of the form there is question A: a radio-type question (Line 5), and question B which has type “none” (Line 6). Question B contains an inline, repeating question list (Line 7),^13^ featuring three, non-numbered text-type questions (Line 8). As it was in our case (in Fig. 6), one might want to know several aspects about a particular entity, such as patient conditions: when each condition was diagnosed, by whom, its ICD code, and so on. In this scenario, it makes little sense to repeat each question individually, but rather it would be preferable to repeat the whole set of questions in an ‘inline’ fashion, simulating a table.

**Fig. 7:**
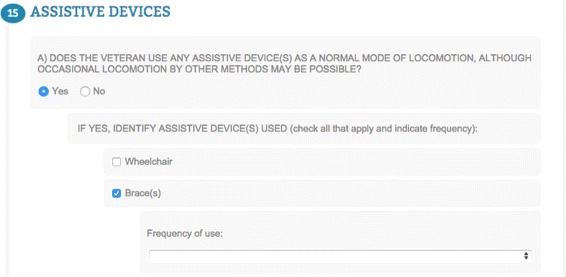
Form question with hidden sub-questions that get triggered depending on the selected answer. Question on assistive devices consisting of a first question whose “Yes” answer triggers sub-questions. Similarly, when an assistive device is selected from the list, a sub-question appears prompting the user for the frequency of use of the respective device

Question A of Section 15 (see Fig. 7) is configured so that when the option “Yes”, corresponding to the OWL individual *cfa:Yes*, is selected (Line 9), it triggers the appearance of its sub-questions; a list of assistive devices, each of which when selected, triggers the appearance of a sub-question relating to the frequency of use of that particular assistive device.

**Fig. 8:**
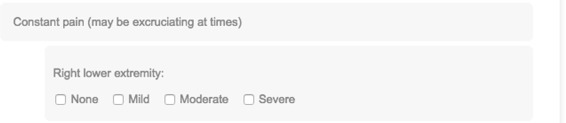
Question with custom order of answer options. DBQ question where the default, alphabetical order of answer options is overridden in the configuration

In Fig. 8, under ‘Right lower extremity’, we have a question with a list of answer options derived from an enumerated value set, which would ordinarily be ordered alphabetically. However, ‘None’ would then appear between ‘Moderate’ and ‘Severe’, thus interrupting a severity scale. So we added: *optionOrder=*“3;*” to the *question* element in Line 16 of Code Snippet 1, which states that the third option (or would-be third option alphabetically) should appear first, and the remaining should be presented in default order.

### Analyzing DBQ Submission Data

One of the authors (Michael J. Tierney), who is a physician from the VA Palo Alto Healthcare System, verified the generated OWL-based version of the DBQ form throughout its development, to ensure that, for example, a particular question triggers the correct set of sub-questions when a specific answer is selected. This was done by manually inspecting the original Portable Document Format (PDF) version of the DBQ form, and comparing it with the generated Web form (taking into account the extended capabilities of our system).

MJT filled in the “Back (Thoracolumbar Spine) Conditions” DBQ with 5 complete sets of sample patient data. The data gathered are stored in a graph database with support for SPARQL 1.1 querying and OWL 2 reasoning based on the Pellet reasoner [17]: Stardog v2.2.4.^14^

Since our data are both structured and semantically enriched, we are able to query the observations using SPARQL, classify them into criteria representing powerful OWL expressions, or manipulate them using SWRL. For example, Code Snippet 2 presents a simple SPARQL query that returns all instances of *Observation* where a patient presented signs or symptoms due to radiculopathy. It is worth observing that this query is formulated in such a way that it is independent of the assessment instrument, including the particular formulation of the question, but rather uses the appropriate focus individual from our CFA ontology.



In order to query for all observations of severe pain anywhere in the lower extremity, one could formulate an OWL DL query such as that given in Code Snippet 3. The query makes use of the appropriate ICF codes for ‘pain in body part’ and ‘structure of the lower extremity’, while the remainder of the query exploits the instantiation of *Observation* (from the *datamodel* ontology) as well as the clinical functional assessment-related modeling in the CFA ontology for, for example, the pain severity value and anatomical location.



In response to the query in Code Snippet 3, a DL reasoner uses the semantic descriptions of the observation foci, which are derived from the *isAbout* property of questions, to aggregate answers for severe pain for different parts of the lower extremity from all submissions of potentially different forms.

## Discussion

In this paper, we presented a system for OWL-based form generation and data acquisition, which gathers form answers as tab-delimited data, RDF triples, or OWL instances that can be subsequently analyzed in a systematic way (as shown in our queries in the Analyzing DBQ Submission Data Section). Once the raw data is processed (by deriving the foci of observations from the *isAbout* field of the questions), the resulting data have no dependency on specific questions (except for provenance tracking), so even if the form specification is modified, previous form data are still comprehensible and sound (i.e., upon form specification changes the new data and old data remain compatible). The value of data in such a structured format in any arbitrary domain is twofold: automating, or improving the automation of the process of arriving at desirable conclusions from questions in the form using Web standards, and for further analysis and querying of data associated with ontologies. For example, the acquired data can be readily checked for consistency with the associated ontologies.

In the clinical functional assessment domain, our modeling of forms and questions is consistent with the format of assessment instruments defined in LOINC [18]. However, the types of queries we formulated for functional assessment data are unfeasible using LOINC, since LOINC provides no semantics behind what an answer to a specific question means. Similarly, LimeSurvey,^15^ a Web application for building and managing online surveys and databases, and REDCap [19], a similar tool commonly used in clinical research, do not provide ontology-based descriptions of their data elements. Consequently, these tools cannot directly support queries that make use of Semantic Web technologies. Nevertheless, given the popularity of these existing tools, it is important to investigate how to annotate metadata that define these instruments with ontology terms [20]. Such annotations are analogous to the use of the *isAbout* property in our representation. The recent work of Jiang et al. [21] to create description logic expressions from definitions of cancer study common data elements (CDEs) is a first step to endow CDEs, which are commonly used in clinical research tools and standards, such as REDCap and Clinical Data Interchange Standards Consortium (CDISC), with ontology-based annotations. Jiang et al. created such expressions by using the SNOMED CT observable model [22] and the NCI Thesaurus [23, 24]. We can also create such definitions in the work presented here, using the datamodel and domain ontologies.

We presented our modeling of functional assessments and assessment instruments, and demonstrated (1) how to generate forms and acquire data based on these OWL ontologies and data models, and (2) how to make use of the data using queries on individual subjects and queries that aggregate population data. The modeling contributions include (1) *datamodel*: an information model that allows the specification of generic assessment forms and the format of structured data acquired through the instruments, and (2) *CFA*: a clinical functional assessment domain ontology that allows defining questions being asked in an assessment instrument in terms of a rich ontology that integrates standard terminologies such as ICF and SNOMED CT, and provides the means for making detailed or aggregate queries on acquired data.

We have designed our output model to support the acquisition of structured data through Web forms, and for the potential to integrate the data inside EHRs. It is straightforward to transform the data we capture as instances of *Observation*, *Certification*, *EvaluatorInformation*, and *SubjectInformation* into, for example, Health Level Seven (HL7) Reference Information Model (RIM) standard compliant data [25]. Finally, we have shown that the problem of structured data acquisition can be suitably tackled using OWL; our solution, though applied to the clinical functional assessment domain for the context of this paper, is easily generalizable to an arbitrary domain.

In the future, we plan to build a user interface that would allow users to configure a form without any knowledge of XML—currently a potential obstacle for wider adoption. This user interface would reuse the form generation engine presented in this paper. The current implementation of the form generator could benefit from more UI diversity, for example, by providing more than one CSS style for the forms. There are other minor aspects of the tool configuration that we plan to enhance, for instance, the ability to overwrite the text of a question.

## Conclusions

We presented a tool for generating Web forms based on OWL ontologies, and acquire instance data that relates to ontology terms with potentially complex class expressions associated with them. We use ontologies to specify the structure of Web-based forms and the structure of data to be acquired from the forms, and to semantically enrich the data elements in the forms. The system we developed, applied here to clinical functional assessment, can be easily adapted for any other use case. Using our system, users can structure and acquire data in formats that are highly amenable to querying as well as reasoning, and therefore can be more easily analyzed and validated. Data elements in the forms, such as “severity of constant pain caused by radiculopathy in the lower right extremity”, are not represented as opaque tokens, but have detailed descriptions in the domain ontology. As a consequence, the data acquired through the forms are fully integrated into the ontologies. This integration enables inferences and queries on the data that are otherwise not feasible. Additionally, the consistency of the input data against the domain ontologies can be verified prior to, or upon submission of forms.

## Endnotes

^1^ https://github.com/information-artifact-ontology/IAO

^2^ The typical kinds of schema accepted are OWL and RDFS.

^3^ http://www.w3.org/TR/rdf-sparql-query

^4^ http://owlapi.sourceforge.net

^5^ http://github.com/protegeproject/facsimile

^6^ http://www.eclipse.org/jetty

^7^ http://ant.apache.org

^8^ https://github.com/protegeproject/facsimile/tree/master/ontology

^9^ http://www.who.int/classifications/icf/en

^10^ http://www.ihtsdo.org/snomed-ct

^11^ http://www.vba.va.gov/pubs/forms/VBA-21-0960M-14-ARE.pdf

^12^ Radiculopathy is an irritation of or injury to a nerve root that typically causes pain, numbness, or weakness in the part of the body which is supplied with nerves from that root.

^13^ For presentational purposes, the third repetition is omitted in Fig. 6.

^14^ http://stardog.com

^15^ https://www.limesurvey.org

## Abbreviations

CFA: Clinical functional assessment
CSV: Comma separated values
CSS: Cascading style sheets
DBQ: Disability benefits questionnaire
DL: Description logic
DTD: Document type definition
EHR: Electronic health record
HTML: Hyper text markup language
HL7: Health level 7
IAO: Information artifact ontology
ICF: International classification of functioning, disability and health
ICD: International classification of diseases
IRI: Internationalized resource identifier
PDF: Portable document format
RDF: Resource description framework
RDFS: Resource description framework schema
RIM: Reference information model
SPARQL: SPARQL Protocol and RDF Query Language
SWRL: Semantic web rule language
SNOMED CT: Systematized nomenclature of medicine –clinical terms
LOINC: Logical observation identifiers names and codes
OWL: Web ontology language
VA: United States Department of Veterans Affairs
W3C: World wide web consortium
WHO: World Health Organization
WHO–FIC: World health organization family of international classifications
NCBO: National Center of Biomedical Ontology
XML: Extensible markup language
